# Level of play and coach-rated game intelligence are related to performance on design fluency in elite soccer players

**DOI:** 10.1038/s41598-020-66180-w

**Published:** 2020-06-25

**Authors:** T. Vestberg, R. Jafari, R. Almeida, L. Maurex, M. Ingvar, P. Petrovic

**Affiliations:** 1 Department of Clinical Neuroscience, Karolinska Institutet, Stockholm, Sweden; 2 Stockholm University, Brain Imaging Center, Stockholm, Sweden

**Keywords:** Attention, Human behaviour

## Abstract

Executive brain functions are innate mechanisms for regulating behavior. While the impact of suboptimal executive functions has been characterized in patients, their contribution to individual success has not been elucidated. We set out to understand how executive functions relate to successful human behavior by examining their relation to game intelligence in sport - the ability to read a game and quickly adapt the behavior. In elite soccer players (n = 51), those playing in national teams (national team players) significantly outperformed those only playing at premier league level (premier league players) in *Design Fluency* (*DF*), a complex visuo-spatial executive function test that includes measures of creativity and cognitive flexibility. Their result showed a moderate correlation with coach rated game intelligence, remained also when correcting for low level cognitive capacity and was most evident when considering cognitive flexibility. *DF* capacity also correlated with number of assists made during the season but not with number of made goals during the same period, linking the fast planning of several steps in *DF* to fast planning of several steps in the soccer game. Altogether, our data suggests that *DF* capacity relates to success in soccer both on a subjective and on an objective level.

## Introduction

Dynamic adaption of behavior is a prerequisite for reaching an overall goal in a constantly changing environment. To enable such adjustments, a set of specialized cognitive processes that regulate behavior have been favored in evolution^1^. These mechanisms, often called executive functions (EF), are associated with behaviors such as response inhibition, multiprocessing, cognitive flexibility, and are closely related to working memory and creativity^1–5^. EF are highly dependent on prefrontal and parietal brain regions that work in tandem with basal ganglia and specific neuromodulatory systems^5–11^. Tests of EF have been used for decades to assess frontal lobe damage as well as neuropsychiatric disorders in patients^5^. Although EF have important implications on problem solving they are sparsely related to IQ^12^. However, while high scores in IQ and general mental ability seem to predict individual achievement in several domains^13,14^, the importance of EF for success has been less studied.

Ball sports require continuous adaptive behaviors and should therefore also involve general cognitive processes such as EF. In order to understand how EF relate to successful behavior in real life we have previously assessed elite soccer players^15,16^. We chose soccer as the behavior is constrained in terms of rules, spatial extent and time, and hence, well-suited to study scientifically. Moreover, soccer represents a global cultural phenomenon with 265 million active players worldwide^17^ and understanding the underlying mechanisms is therefore of a general interest.

Acquired domain-specific behavioral and visuo-perceptual skills are important in elite sports^18,19^ including soccer^20,21^. Arguably, also more general cognitive processes should be decisive for a successful behavior in these contexts. For example, a successful elite soccer player needs to process a large amount of information in a short time under mental pressure, and be able to quickly adapt, change strategy and inhibit responses. Elite players often demonstrate creative decision-making with a large degree of accuracy at high speed^22,23^. These observations suggest that successful elite soccer play requires extraordinary EF in several domains that can orchestrate the learned domain-specific skills - as has been proposed for any type of complex behavior^11^.

In line with this hypothesis, we have previously shown that adult elite soccer players have significantly better EF compared to sub-elite players^15^. Following this study, a similar link between EF and level of play has been suggested in several soccer studies^16,24–28^ as well as in studies of other ball sports^24,29–38^. As some studies have not identified a difference in EF between elite and semi-elite players in ball sports^39,40^ this question is still under debate. Correlational analysis have suggested that EF scores also predict the number of made goals and assists during a period of 2.5 years in elite and sub-elite players when controlling for age, position and level of play^15^. Similarly, a correlation was found between EF results and made number of goals during two seasons in young elite soccer players^16^. Finally, EF results could partially predict who would be accepted into an academy for young soccer players^26^.

EF may be divided into ***core executive functions (CEF)*** consisting of the separate EF-components such as behavioral inhibition, interference control, aspects of working memory and cognitive flexibility as well as ***higher order executive functions (HEF)*** associated with the use of various EF-components simultaneously and problem solving^3,16^. The importance of different CEF for soccer play is evident, e.g. inhibition of behavior as a response to a feint or the use of working memory to remember the positions of other players on the field. However, soccer behavior in adult elite players is primarily characterized by complex and fast problem solving involving several aspects of EF, suggesting that HEF are fundamental for success. Although many EF components are active simultaneously as a part of HEF, it is possible that some have more impact on successful behavior than other. We have previously suggested that a combination of divergent and convergent creativity under time pressure, important for choosing one out of many possible solutions quickly, is such a key component for successful soccer play. This behavior should also be associated with an excellent cognitive flexibility allowing a dynamic adaption to fast changes on the soccer field^15^. Given the reasoning above, testing only specific CEF would not fully mirror soccer behavior. Instead, more complex tests of HEF involving all the discussed aspects above are warranted. ***Design Fluency*** (***DF***) is a test that involves fast problem solving and creativity as well as working memory and behavioral inhibition. Moreover, it is a visuo-spatial test requiring behavioral responses. Thus, it consists of many components that are theoretically important in soccer behavior. So far, four studies have previously suggested that higher level of soccer behavior is associated with better results on *DF*^15,16,25,26^ including two studies predicting more successful behavior (discussed in the previous paragraph)^15,26^.

Based on the theoretically derived cognitive requirement for successful behavior in soccer discussed above we suggest that HEF are closely associated to the level of *game intelligence* – a term used in ball- and team sports describing a players ability to read the game, quickly adapt and always be at the right place^22,23^. It has been suggested that game intelligence may be a key component for success in elite soccer, as physical skills and coordination alone have a low predictive value^22,23^.

In the present study we first hypothesized that the difference in *DF* results depending on the level of play^15,16,25,26^ should also be observed when only studying premier league players. We therefore tested 51 elite soccer players and compared a group that has been chosen to play for a national team, and thereby shown extra-ordinary quality, with the rest of the players. A second aim of the study was to assess whether their result on *DF* was related to successful behavior. It may be argued that number of goals and/or assists, as measured in our previous studies^15,16^, is not an ideal proxy for game intelligence or successful soccer behavior since various positions may have different main behavioral objectives such as defending the goal or tactically distributing the ball holding. In order to more precisely assess the relation between HEF and game intelligence we instead related an independent assessment of game intelligence with the players *DF* results. The assessment of game intelligence was performed by the main coach in the studied teams. We hypothesized that if EF are truly associated with game intelligence there should be a significant relationship between the variables. Finally, we assessed whether the *DF* results were related with objective measures such as number of assists and goals.

## Results

### Overview of the current study

Since soccer is complex in terms of speed and control of information processing, as well as decision making per time unit, a specific demand on the executive system seems to be important for success^16^. Instead of only single core EF (CEF) - including response inhibition, working memory and set shifting – such behavior requires the use of several EF simultaneously involved in problem solving, creativity and planning, sometimes denoted as higher order EF (HEF)^3,16^. Our prime test of such demanding HEF was *Design Fluency* (*DF*)^41–43^ from the Delis-Kaplan Executive Function System test battery (D-KEFS)^41,42,44^ since it both involves visuo-spatial information processing and includes planning, creativity, working memory, response inhibition and cognitive flexibility – mirroring the complex soccer game on an information processing level (see Methods). As in previous studies we used *Trail making test* (*TMT*) and *Colour-word interference test* (*CWI*) from the D-KEFS^41,42,44^ as well as a set tests from CogStateSport computerized concussion testing (CS)^45,46^ that captures basic cognitive capacities like processing speed as well as aspects of core executive functions (CEF) including short term and working memory for exploratory analyses. Measuring both CEF and HEF made it possible to study the specific involvement of high executive demand in relation to game intelligence.

In order to control for increase of EF capacity due to physical and/or cognitive training^47,48^ we only assessed elite soccer players belonging to the Swedish premiere league in the present study, as they practice to a similar degree in standardized training schedules. Although training and other contextual factors are similar for premier league players, there still is a difference in quality of play between individual players. One factor that may suggest an exceptional high quality of play is whether the individual player has been selected to play for the national team. We reasoned that a substantial part of this quality would be mirrored in measures of fast and focused information processing capacities such as EF as well as in game intelligence. We therefore compared players that had been selected for national teams (national team players, NTP) with those players that have only played in the Swedish premier league (premier league players, PLP) and reasoned that NTP should show both a higher degree of HEF and game intelligence than other elite players (PLP).

The included subjects belonged to the four Swedish premier league teams and consisted of both male (n = 19; Mean age=24.4, SD = 4.73) and female (n = 32; Mean age= 24.5, SD = 4.63) players. Out of the 51 players 23 had previously played for fourteen national teams on senior level.

### *Design Fluency* – Group effects

**Figure 1:**
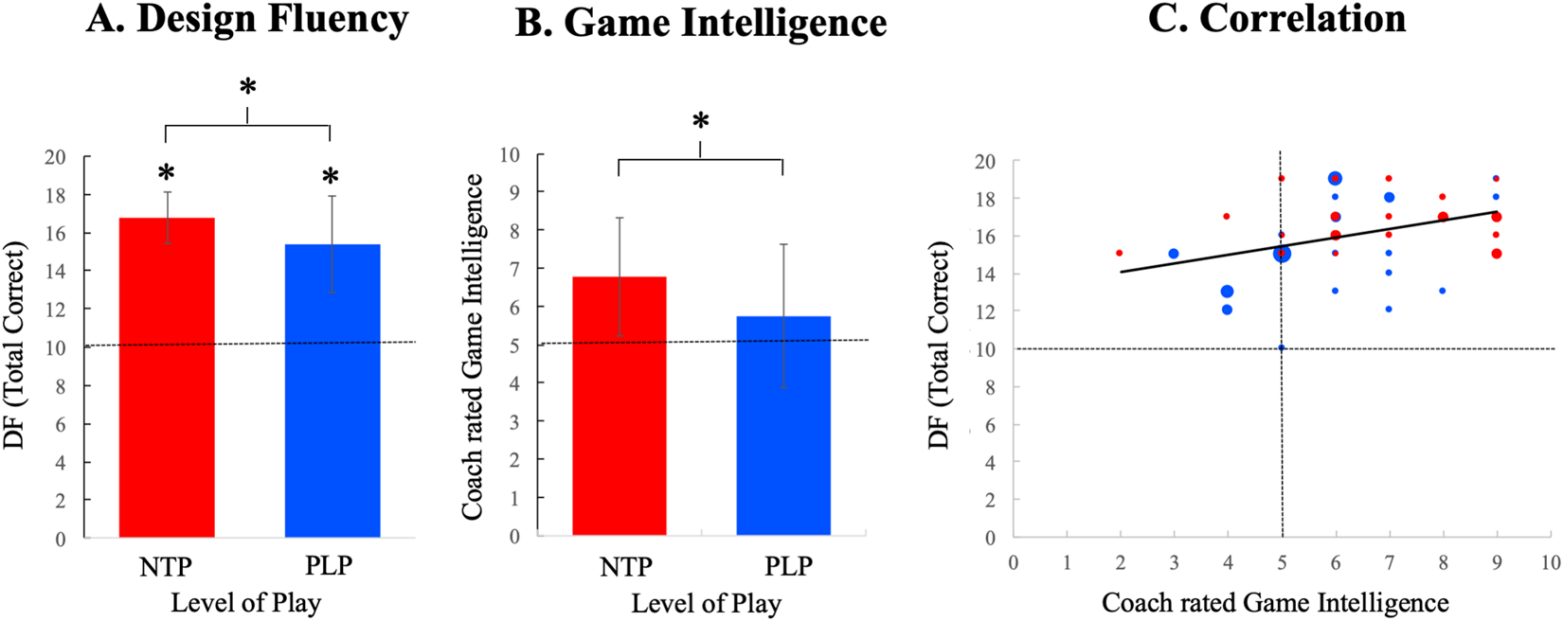
(**A**) While NTP (red) and PLP (blue) were significantly better than the norm (dotted line) in *Design Fluency* (*DF-total correct*; NTP: Mean=16.74, SD = 1.36, *t*(22) = 23.84, *p* < 0.001, *d* = 4.97 and PLP: Mean = 15.36, SD = 2.54, *t*(27) = 11.15, *p* < 0.001, *d* = 2.11), NTP were significantly better than PLP (*t*(42.67) = 2.48, *p* = 0.017, *d* = 0.75). Thus, both our previous study^15^ and the present result suggest that elite soccer players are approximately 2 SD better than the norm in this test of EF (3 points on the normalized scale equals 1 SD). The difference between NTP and PLP remained significant when performing an ANCOVA correcting for reaction time, CEF represented by one-back working memory task, aged and sex (F(1,45)=5.96, p = 0.019, d = 0.73). **(B)** Coach ratings suggested that NTP had significantly larger game intelligence than PLP (NTP: *Mean* = 6.78, *SD* = 1.88; PLP: *M* = 5.75, *SD* = 1.56 *t*(49) = 2.15; *p* = 0.035, *d* = 0.66). Although the data was normally distributed and therefore we used a parametric test, the significant result remained if using a non-parametric test (*p* = 0.004). **(C)** *DF-total correct* was significantly correlated with Game Intelligence (*r* = 0.37, *p* = 0.008). The *r*esult remained significant when performing a more conservative analysis correcting for level (NTP or PLP) (*r* = 0.3, *p* = 0.032). NTP = National Team Players (highest level of play); PLP = Premier League Players (highest level of play);.

Firstly, we showed that both groups were far better than the norm in *DF-total correct* (i.e. total number of correct designs in *DF*; Fig. 1A) as well as other EF-tests (exploratory analyses are presented in Supplementary Tables 1–10) reproducing our previous results of adult elite players^15^. In line with our hypothesis, NTP were significantly better than PLP in *DF-total correct* (*t*(42.67)=2.48, *p* = 0.017, Cohen’s *d* = 0.75). In order to assure that the results were not confounded by other factors we also performed an ANCOVA-analysis in which we controlled for several variables including: 1) cognitive process-speed (to assure that the effect was not due to general faster response speed), 2) simple working memory using a one-back working memory task (used as a proxy for CEF), 4) sex and 5) age. This model still showed that NTP had significantly higher scores than PLP on *DF-total correct* (F(1,45)=5.96, *p* = 0.019, Cohen’s *d* = 0.73; Supplementary Table 1). There was no significant interaction between sex and level of play (p = 0.20) and the interaction effect was therefore not included in the final analysis model. The result suggests that reaching international player level does not only pertain to general cognitive capacity (including processing speed and CEF) but can also be ascribed to more taxing information processing as mirrored in a high demand on the EF system (including HEF).

### Relation between *Design Fluency* and coach rated game intelligence

While there is no established exact definition of game intelligence^22,23^, professional coaches in the highest elite teams have a strong and uniform opinion of its components and level in individual players. We therefore reasoned that their ratings may be a proxy measurement of game intelligence level in the individual players. The coaches were therefore asked to rate the level of game intelligence for each player of the team using a scale with three anchors that relates to players in the Swedish premier league (1: lowest possible game intelligence; 5: game intelligence of an average player; 9: highest possible game intelligence). Not surprisingly, our result showed that NTP had significant higher coach rated game intelligence than PLP (Fig. 1B).

A main analysis in the present study was to better understand whether game intelligence is related to *DF*. A correlational analysis between coach rated game intelligence and *DF-total correct* demonstrated a highly significant and moderate relation between the two factors (*r* = 0.37, *p* = 0.008). The result remained significant when analyzing the data with a non-parametric test (CC:0.39, p = 0.004). A possible confound could be that professional coaches systematically ascribe higher game intelligence to NTP. Therefore, we also performed a conservative statistical test that corrected for level of play (NTP or PLP) in a partial correlation analysis. The correlation between *DF-total correct* and rated game intelligence remained significant (*r* = 0.3, *p* = 0.032). For transparency reasons we performed exploratory analyses that showed no significant relation between rated game intelligence and Stroop test or working memory (see Supplementary Table 11).

### Subcomponent analysis of *Design Fluency*

**Figure 2:**
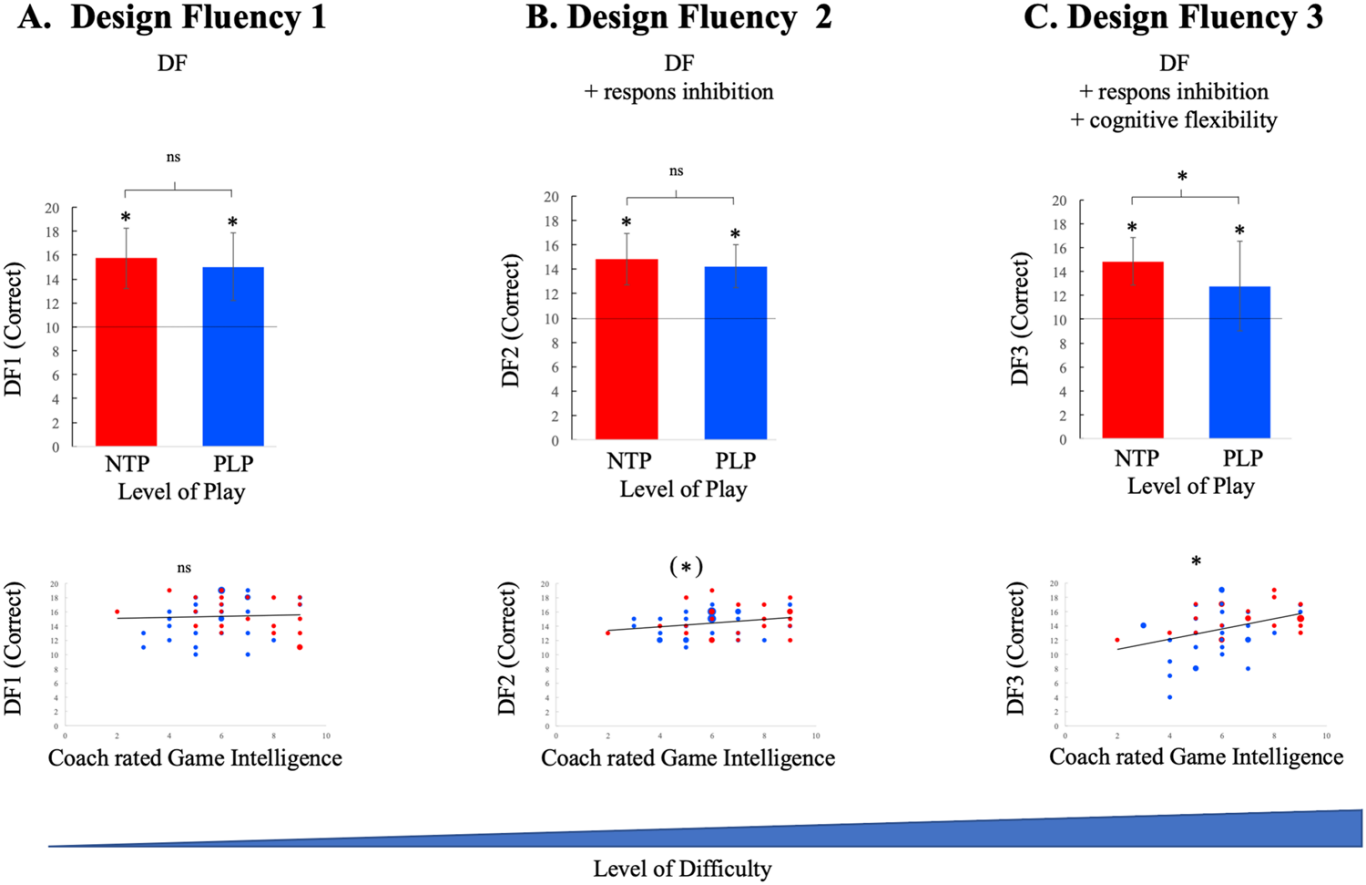
(**A**–**C**) NTP (red) and PLP (blue) were significantly better than the norm (dotted line) for all *Design Fluency* subtest including *DF1* (NTP: Mean = 15.74, SD = 2.51, *p* < 0.001; PLP: Mean = 15.04, SD = 2.83, *p* < 0.001), *DF2* (NTP: Mean=14.83, SD = 2.10, *p* < 0.001; PLP: Mean = 14.25, SD = 1.76, *p* < 0.001) and *DF3* (NTP: Mean = 14.83, SD = 1.99, *p* < 0.001; PLP: Mean = 12.79, SD = 3.765, *p* = 0.001). While there was no significant difference between the groups in *DF1* or *DF2* (*DF1*: t-value= 0.93, *p* = 0.36; *DF2*: t-value = 1.07, *p* = 0.29), NTP were significantly better than PLP in *DF3* (t-value = 2.48, *p* = 0.017, equal variance not assumed). Similarly, while there was no significant correlations between result and coach rated game intelligence for *DF1* (*r* = 0.047, *p* = 0.75) or *DF2* (*r* = 0.24, *p* = 0.09), there was a significant correlation between result and coach rated game intelligence for *DF3* (r = 0.39, *p* = 0.004). This effect remained significant when correcting for *DF1* and *DF2* in a partial correlation analysis (r = 0.352, p = 0.013). NTP = National Team Players (highest level of play); PLP = Premier League Players (highest level of play); *DF* = *Design Fluency*.

A strength of a composite measure of *DF* is that it mirrors many cognitive requirements of soccer behavior. In order to pinpoint the major sub-components of HEF relevant for successful behavior an analysis of different sub-test was performed. The three different subtests in *DF* (*DF1*, *DF*2 and *DF*3 – *correct responses*) increase in cognitive requirement for each level of *DF*. While *DF1* contains the fundamental aspects of *design fluency* including working memory and creativity, *DF2* adds an extra component of response inhibition and *DF*3 adds cognitive flexibility (see Methods). We therefore performed several exploratory analyses on the different *DF* subtests. We analyzed 1) difference between the two groups (NTP and PLP) and the norm, 2) difference between NTP and PLP as well as 3) the relation between *DF* capacity and coach rated game intelligence. While we observed that NTP and PLP were significantly better than the norm for all subtests (Fig. 2), there was no significant difference between the groups in *DF1* or *DF*2 (Fig. 2A,B). Moreover, there was no significant correlation between the result and coach rated game intelligence for *DF1* and *DF2* - although the latter suggested a trend effect (*r* = 0.24, *p* = 0.09). However, at the highest demand level in *DF*3 we observed a significant difference between the groups with a better result for NTP than PLP (t-value=2.48, *p* = 0.017) – Fig. 2C. The result of *DF3* also significantly correlated with coach rated game intelligence on a moderate level (*r* = 0.39, *p* = 0.004). Performing a partial correlation suggested that this effect remained when correcting for *DF1* and *DF*2 (*r* = 0.352, *p* = 0.013). These results suggest that either cognitive flexibility or general increased task demand may be specifically associated with game intelligence. However, as suggested in Fig. 2 it is not possible to exclude that ceiling effects in *DF1* and *DF2* also may explain the findings.

### Speed and accuracy

**Figure 3:**
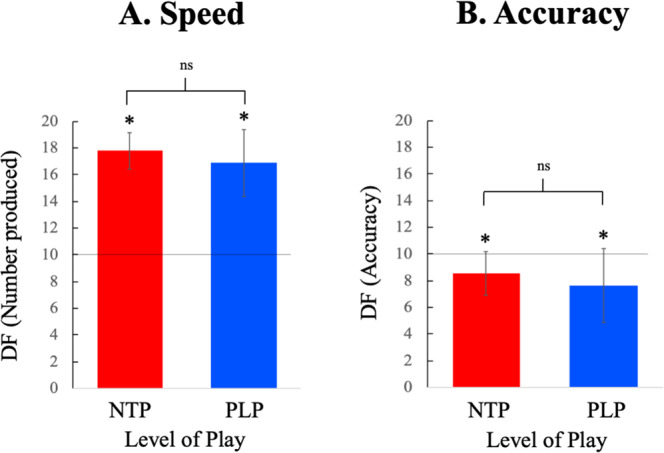
While both NTP and PLP were significantly faster than the norm (measured by number of produced designs) there was no significant difference between the groups (NTP: Mean=17.78, SD = 1.38, t-value = 27.04, *p* < 0.001; PLP: Mean= 16.89, SD = 2.50; t-value = -14.59, *p* < 0.001; NTP vs PLP: t-value = 1.61, *p* = 0.12). While both NTP and PLP were significantly less accurate than the norm there was no significant difference between the groups (NTP: Mean = 8.57, SD = 1.62, t-value = -4.25, *p* < 0.001; PLP: Mean=7.64, SD = 2.74; t-value = -4.56, *p* < 0.001; NTP vs PLP: t-value = 1.49, *p* = 0.14).

Apart from delivering a high number of correct responses when solving a cognitively demanding creativity visuo-spatial task, the elite soccer players were also extraordinarily fast (Fig. 3A). The cost for being fast was that they were slightly less accurate than the norm (in between 0.5 and 1 SD; Fig. 3B). Although, there was no significant differences between the groups in these measures NTP tended to be both somewhat faster and somewhat more accurate in *DF* than PLP, a combination that may explain the main result.

### Relation between *Design Fluency* and goals or assists

Finally, we wanted to test whether the *DF* results also were related to an objective measurement of behavior (in contrast to coach rated game intelligence) as suggested in our previous study^15^. In order to do that we studied number of made goals and assist for the season 2017–2018 (divided by number of played matches) as proxies for successful behavior in players that have actively played games during that period (n = 48). We separated goals and assists, as assists are theoretically more dependent on predicting a subsequent behavior made by the next player (compared to made goals). We then performed an ANCOVA analysis where we adjusted for age and position as done in our previous study^15^. We also controlled for sex (as both men and women were part of the present study) and low level cognition (cognitive process-speed and simple working memory using a one-back working memory task) to specifically study HEF. In line with our hypothesis we showed that there was a significant correlation between assists and *DF-total correct* (F(1,40)=4.94, p = 0.032 Cohen’s *d* = 0.70). This correlation was even more pronounced for players that had performed at least one assist (which ensures that they are in position to produce assists) (F(1,20)=9.83, p = 0.005, Cohen’s *d* = 1.4; assist were square root transformed in this analysis to achieve a normal distribution). In contrast, we observed no such effect between *DF-total correct* and made goals using the same ANCOVA (F(1,40)=0.49, p = 0.49, Cohen’s *d* = 0.22).

## Discussion

While it is known that EF predicts behavior in psychiatric disorders and frontal lobe dysfunction in the low end, its relation to behavior at the high end has not been fully explored. Our results suggests that elite soccer players that have played in national teams perform better in *design fluency* (*DF*) than those that have only played in a premier league. Moreover, a professional third person (head coach of the team) estimate of a players’ game intelligence - based both on a long experience of successful behavior amongst elite players in general and on the tested individual in specific - was substantially explained by *DF*. Apart from subjective rating of game intelligence made by the coach, we also showed that an objective measurement of successful soccer behavior, i.e. number of assists, correlated with *DF* capacity. This strengthens our initial findings since it suggests that fast planning of different designs in the *DF* test relates to fast spatial planning in several steps when playing soccer. In contrast, number of made goals, that only involves planning of one step, did not correlate to *DF*. In our previous study where we showed a relation between *DF* and behavior we did not separate goals and assists^15^. Here, we show that the effect is driven by assists in line with the theoretical considerations (at least in premier league players).

We used *design fluency* as our prime test of EF given that it mirrors the behavior on the soccer field in terms of creative planning, inhibition and cognitive flexibility. A criticism against using *DF* is that it is a task involving several EF-components and cannot easily isolate one single process. However, this also better mirrors real life demands, where a single EF-component is seldom in used in isolation. The involvement of problem solving and the use of various EF-components at the same time has been termed higher order EF (HEF)^3,16^ and has not been explored extensively. The present result suggests that HEF relates to complex real-life behavior. However, performing an analysis of *DF* subtests (*DF1*, *2* and *3*) suggests that cognitive flexibility may have special impact on the difference between NTP and PLP as well as the relation to coach rated game intelligence. Alternatively, the results may suggest that NTP are better than PLP in more difficult tasks. Also, the effects may be explained by ceiling effects in *DF 2* and *3*. Interestingly, the only difference between NTP and PLP in *Color word Interference* (*CWI*) a D-KEFS variant of the Stroop test^41,42,44^ was *CWI 4* - a Stroop test component involving cognitive flexibility emphasizing the importance of this cognitive function at this level of play (Supplementary Table 10). Exploratory analyses showed no significant relation between rated game intelligence and *CWI* or WM. However, the results do not preclude that other EF-components, including inhibitory control and working memory, have a decisive role for successful soccer behavior. Future studies with a larger power and more specific tests could better elucidated their contribution to game intelligence in soccer.

Our results adds to previous studies pointing towards that ball sports played at higher level^15,16,24–38^, including elite soccer^15,16,25–28^, are associated with better EF than when played at lower level. Also, the results are in line with two meta-analyses that compared elite with non-elite athletes (including those playing ball sports) in more broadly defined general cognitive task including low level attentional tasks^49,50^. A key question is why this difference is observed. We postulate that a main reason is that the complexity of the play requires above normal HEF. However, there are other possibilities as well. For example, cognitive^47^ and physical^48^ training may improve EF. Although we see that as an unlikely reason for the present result, as NTP and PLP are subjected to the same standardized training procedures, it cannot be excluded. While both broad and narrow transfer effects may occur in sport from training^51^, the impact of transfer in general cognitive functions has been debated^47,52^. Also, although increased cognitive functions are readily observed in all elite athletes preliminary studies suggest that those playing ball sports outperform other athletes^37,53^. Future studies are warranted to better understand how EF related specifically to ball sports.

A related question is whether a soccer player needs to have well-developed HEF to be able to play at an elite level or whether it is a consequence of HEF-development after long-term play on an elite level. Large longitudinal studies would be necessary for understanding this relation better, but we our initial hypotheses is that both aspects are relevant.

There are several limitations in the present study. First, although we hypothesize that HEF are key factors for a successful soccer behavior and our results are in line with this idea the study cannot prove causality. Adjusting for different specific confounding variables (such as physical ability, basic skills and motivation) in future studies may shed more light on this question. Another limitation concerns the norms used in the present study, that are relatively old and based on a US population-sample^41^. It is possible that EF capacity in the population has changed since the norm was constructed. However, the norm is based on a larger population than many other EF tests, and we note that the present results on elite soccer players are in line with our previous study where the players were tested 2007^15^. Sampling and testing of the participants also may be viewed as a limitation. In two of the teams almost all players chose two participate and difference between NTP and PLP was still significant using the same analysis as above (p = 0.041). There is a possibility that NTP players were differently treated as compared to PLP, although we regard this as unlikely as the tester used standardized instructions. Finally, this is a small and preliminary study with several measurements that have not been used previously such as coach rating of game intelligence. Although this rating has face validity, it also suffers from not having been assessed for reliability or validity, especially since several raters were involved, i.e. one coach rated all subjects in each one of the four teams. Adjusting the model for the four coaches rating the players would be an overly conservative approach since it also adjusts for difference between the four premier league teams that were included in the study. Thereby it would also control for the variable if interest, i.e. better DF capacity and game intelligence that is present in the better teams. Nevertheless, we performed such an analysis for transparency and it still suggests that there is a relation between rated game intelligence ratings and design fluency results on a threshold level for *DF-total correct* (F = 3.07, p = 0.086) and on a significant level for *DF3* (F = 5.22, p = 0.027). For the main analysis we have to rely on the fact that the ratings are somewhat consistent between the coaches as they had been active in the same premier league for a long time and the rating scale had specific anchors related to players in the Swedish premier league. In future studies measurements of inter-rater correlations would be of importance. Other methods such as using peers to rate each other would also be possible alternatives.

Ball sports require continuous adaptive behaviors in order to perform successfully and are constraint in terms of rules, space and time. In fact, a spatially defined area in which freely moving human agents try to achieve different objectives resemble testing conditions that have successfully been set up to study experimental animals^54,55^. In other words, team ball sports constitute a perfect condition to study detailed human behavior. Here, we make an initial such step by suggesting that HEF may relate to measured success in soccer both on a subjective and on an objective level.

## Methods

### Ethics statement

The study was approved by the local ethical committee (Regionala etikprövningsnämnden i Stockholm; Dnr 2017/2453-31/5) and was performed in full compliance with the Declaration of Helsinki. All subjects were given verbal and written information about the study and gave their verbal and written informed consent to participate.

### Participants

Four teams in the Swedish premier league (Allsvenskan) accepted that their players were tested for the present study. The players in the four teams were then asked whether they would like to take part in the study and 51 players accepted to participate (19 men and 32 women; age range 17 to 35 years, Mean=24.5 years, SD = 4.6; Men: age range 18 to 35 years, Mean=24.5 years, SD = 4.7; Females: age range 17 to 33 years, Mean=24.5 years, SD = 4.6). In this group of 51 players, 28 players had never played for a national team on senior level (denoted as Premier League Players, PLP) while 23 of the players had played at least one game for a senior national team (denoted as National Team Players, NTP). The 23 NTP had played for 14 different national teams around the world. There was a significant difference in age between NTP or PLP (NTP: The age range 21 to 35 years, Mean=26.8 years, SD = 4.1; PLP: The age range 17 to 33 years, Mean=22.6 years, SD = 4.2; Difference between NTP and PLP: t-value=3.53, p-value=0.001). There were 7 male and 16 female players in NTP (i.e. 30.4% male and 69.6% female players) and there were 12 male and 16 female players in PLP (i.e. 42% male and 57% female players).

### Test material

In the present study we used tests from two test batteries: The Delis-Kaplan Executive Function System test battery (D-KEFS)^41,42,44^ and CogStateSport computerized concussion testing (CS)^45,46^.

D-KEFS is a test battery measuring different aspects of EF^41,42,44^. All tests in the battery are performance tests. Primary measurement is often time in sec or responses per time unit. Secondary measurement is often accuracy. D-KEFS is routinely used in clinical assessments of patients around the world. A test-retest reliability analysis for the D-KEFS tests has been performed on the D-KEFS norm group (1750 individuals stratified on age, sex, ethnicity and education) in US year 2000 and shows a moderate to strong reliability^41^. In general, overall results suggest that most of the test measures possess adequate reliabilities regardless of age groups^44^. D-KEFS tests show a normal distribution in healthy subjects^41,56^ and the result relates to brain morphology changes^57,58^ within networks involved in EF^7^.

CS is a non-verbal psychomotor test battery that measures basic attention, cognitive process speed, decision-making, speed and accuracy of short-term memory and encoding of working memory^45,46^. It has been standardized to a normal population of different age spans and sex. CS has been used in assessment on cognitive function in sport after concussion.

As in our previous studies^15,16^ we used *Design Fluency* (*DF*), one of the sub-test in D-KEFS, as our main test. *DF* is a standardized test which measures on-line multi-processing such as a combination of planning, visual scanning, creativity, response inhibition, updating of the working memory and cognitive flexibility^3,41,43^ and thus simulates the executive chain of decision making that may be relevant for fast and accurate behaviour. It has been suggested that *DF* is a test of higher executive functions (HEF)^16,26^. *DF* is a non-verbal psychomotor test in which the participant uses pen and paper (or a touch screen), i.e. it is not dependent on language skills. In the first condition (*DF1*) the task is to bind together filled dots with four lines under time pressure (60 sec) in order to produce as many different figures as possible. The participant is not allowed to use the same solution twice. Thus, a main aspect of the test is a combination of divergent and convergent creativity. The participant needs to remember previous responses in using working memory and update new rules accordingly (i.e. not repeat previous combinations). He/she must use inhibition skills in order not to repeat previous responses. The participant also needs to constantly use a scanning skill to find new solutions to fulfil the task. The ability to quickly make a strategic plan should also have a large impact on how many designs the subject will be able to make. In the second condition (*DF2*) unfilled dots have been added to the square, and the task is to combine them with lines as in *DF1*. The filled dots are still present but the participant is not allowed to use them in the task. Thus, this task raises the general level of difficulty through higher requirements of response inhibition. In the third condition (*DF3*) both filled and unfilled dots are present. The task is to connect lines as above but also to constantly switch between a filled and an unfilled dot. Thereby, the task difficulty increases by adding cognitive flexibility.

*DF-total correct* is a combination score consisting of all the correct responses from the three *DF* subtests expressed in RAW-scores as well as in normalized scores according to age and sex (used in the present analysis). It captures both more “simple creativity” and “advanced creativity” (with a higher demand on both inhibition and cognitive flexibility) mirroring the variability of problem solutions needed in when playing soccer.

There is a significant learning effect of the *DF*-test^41^. However, no player had previously performed the test. And, although it cannot be excluded that comparable learning effects occurred while playing games or during training, it is unlikely that this could explain any difference between players as they have similar standardized training schedules (as discussed in the main text).

Apart from *DF-total correct*, the correct responses for each subtask (*DF1*, *DF2* and *DF3*) was calculated and normalized according to age and sex. As the subtests differ in general difficulty as well as in specific cognitive requirements (see above) they can be used to study subcomponents of the involved cognitive capacities: While *DF1* (correct) contains the fundamental aspects of *design fluency* including working memory and creativity, *DF2* (correct) adds an extra component of response inhibition and *DF3* (correct) adds cognitive flexibility. These subtest were used in an explorative analysis of the data (see main text).

As in our previous studies we also used CS tests that captures basic cognitive capacities like processing speed as well as aspects of core executive functions (CEF). In the CS-test the subjects are shown different playing cards on a computer screen and have to react as fast and correct as possible in the various tests using different key responses. In the first test (“*Processing speed*”) - measuring simple response time - the subject has to respond to any card that is displayed. In the second test (“*Attention*”) - measuring simple attention - the subject has to respond whether the card is red or black. In a third test (“*Learning*”) the subject has to respond if he or she has seen the displayed card any time earlier in the test sequences - a measure of more demanding working memory and learning. In the fourth test (“*Working memory*”) - measuring simple working memory / short-term memory - the subject has to decide if the previous card is the same as the card before (i.e. one-back memory-test). We primarily used these test results to correct our main results for low level cognition and CEF.

As in previous studies we used *Trail making test* (*TMT*) and *Colour-word interference test* (*CWI*) from the D-KEFS^41,42,44^ as exploratory tests. *TMT* is a test where the participant is presented with numbers and letters on a paper and has to combine them in a numerical and alphabetic way using a pen. In Condition 2 and 3 the tests combine scanning ability with short-term memory. In Condition 4 the test combine scanning ability, cognitive flexibility, multi-processing and short-term memory. These tests measure core executive functions (CEF), including an alphabetic and numerical aspect, without the creativity or problem solving abilities aspects important in *DF* (HEF). *CWI* is a Stroop test involving verbal inhibition. In test-condition 1 (*CWI 1*) the participant will say the printed color of the squares (green, blue and red) line by line from the top to the bottom of a paper. In test-condition 2 (*CWI 2*) the participant will read the colour words (green, blue and red) in black, line by line, from the top to the bottom of a paper. In test-condition 3 (*CWI 3*) all colour words (green, blue and red) are either printed in the congruent or incongruent colour and the subject should always report the printed colour. The test captures response inhibition and represents the classical form of the Stroop task. Test-condition 4 (*CWI 4*), is similar as *CWI 3* but some words have a frame around them in which the rule reverses (i.e. the subject should report the colour word and not the printed colour). In this test the cognitive demands increase by combining response inhibition with cognitive flexibility. The influence of reading capacity as well as knowledge about alphabetic and numerical order deems *CWI* and *TMT* as not perfect EF tests when the goal is to assess soccer behavior. Therefore, they are not optimal for testing the present hypotheses but used as exploratory analyses as done previously^15,16^.

### Assessment of game intelligence

In order to capture the coaches’ assessment of the players’ game intelligence, the head coaches (one for each tested team, i.e four in total) were asked to judge the players of their soccer team in relation to what they perceive as the average level of the Swedish premier league (Allsvenskan). The coaches used a stanine-scale where 5 is average in the Swedish premier league, 1 is the lowest value, and 9 is the highest value.

### Number of played games, goals and assists

The number of played games for a national team on senior level as well as number of made goals and assists were collected from open data sources.

### Procedure

The players went through the assessment at their teams’ training facilities. The assessment were made from 6 of February to 28 of June 2018. The players were tested in a 40 minutes standardized process with two test-leaders. Analyzing one team where both test leaders randomly tested players, a Mann-Whitney test showed that test leader 1 did not differ in the results of *DF-total correct* from test leader 2 (p = 0.39). Thus, there were no significant differences between the two test leaders.

### Statistical analysis

Data were analyzed using IBM SPSS Statistics 25. Shapiro-Wilks test was used to test distributions for normality. Levene’s test was used to test the homogeneity of variances between the groups. All statistical tests were 2-tailed. The specific tests that were used are listed below.

### Hypotheses testing

Three main hypotheses were tested in the present study: 1) *DF-total correct* results differ between level of play in that NTP perform significantly better than PLP. 2) *DF-total correct* is related to coach rated game intelligence. 3) *DF-total correct* is related to number of assists. One confirmatory analysis was performed: Premier league players (of both levels) have significantly better *DF-total correct* results than the norm. All other analyses are regarded as exploratory.

### Tests that were used for main hypotheses and confirmatory analysis

A one sample t-test was used to compare the results from *DF-total correct* of NTP and PLP with the normal population (D-KEFS defined norm^41^). An independent sample t-test was used to compare the result of *DF-total correct* between NTP and PLP. An ANCOVA was used to compare the results of the two groups after controlling for age, sex, processing speed and simple working memory. As the coach rated game intelligence data was normally distributed and Stanine scale was used (considered as a proxy continuous measurement) a parametric analysis, Pearson correlation, was used for testing whether coach ratings of game intelligence correlated with *DF-total correct*. In order to be transparent we also performed a non-parametric analysis using Spearman’s rho for this analysis. We also used a partial correlation test when analyzing the relation between coach rated game intelligence and *DF-total correct*, but adjusting for level of play (NTP/PLP).

### Tests that were used in the exploratory analyses

**Difference between NTP and PLP in coach rated game intelligence:** An independent sample t-tests was used when assessing whether NTP had higher coach rated game intelligence than PLP. Although normally distributed the test was also performed with a non-parametric analysis using Mann-Whitney test.

***DF1***, ***2*, and** ***3*:** One sample t-test were used when comparing NTP and PLP vs the norm for *DF1*, *2* and *3*. Independent sample t-tests were used to compare the result of *DF1*, *2* and 3 between NTP and PLP. Pearson correlations were used for testing whether coach ratings correlated with *DF1*, 2 and 3. A partial correlation test was used when analyzing the relation between coach rated game intelligence and *DF3* but adjusting for *DF1* and 2.

**Speed and Accuracy:** One sample t-tests and independent sample t-tests were used when analyzing Speed and Accuracy in NTP and PLP.

**Goals:** ANCOVA analysis was used when studying the relation between *DF-total correct* and goals

***TMT***, ***CWI*** **and CogSport tests:** One sample t-tests and independent sample t-tests were used when analyzing *TMT*, *CWI* and CogSport tests in NTP and PLP. Also a Pearson correlation was used when testing for possible correlation between coach rated game intelligence and WM (*Working memory* and *Learning* from CogSport) and Stroop tasks (*CWI 3* and 4 from D-KEFS).

## Supplementary information

Supplementary Information.

## Data Availability

Data that does not disclose individual players are available at 10.5061/dryad.7wm37pvqg.
