# Supplementary material for: Level of play and coach-rated game intelligence are related to performance on design fluency in elite soccer players
Source: Sci Rep. 2020 Jun 25;10:9852. doi: 10.1038/s41598-020-66180-w (PMC7316809; doi:10.1038/s41598-020-66180-w)
Supplement: Supplementary file 1 — Supplementary Information. [file 41598_2020_66180_MOESM1_ESM.pdf]

Supplementary information

Level of play and coach-rated game intelligence are related to performance on design fluency in elite soccer players

Vestberg T, Jafari R, Almeida R, Maurex L, Ingvar M, Petrovic P

Department of Clinical Neuroscience, Karolinska Institutet, Stockholm, Sweden
e-mail: predrag.petrovic@ki.se

Supplementary Table 1. ANCOVA assessing whether there was a significant difference between *DF Total Correct* results of NTP and PLP when correcting for 1) CS processing-speed, 2) CS working memory accuracy, 3) sex and 4) age. We checked that the model assumptions were met using Levene's test of equality of residual variances (p = 0.086) and Shapiro-Wilk test for normality of the residuals ($p = 0.85$).

Source	df	F	Sig.
Sex	1	5.18	0.028
**NTP vs. PLP**	**1**	**5.96**	**0.019**
Age	1	0.09	0.765
CS Processing Speed	1	3.63	0.063
CS Working Memory Accuracy	1	1.49	0.228

Exploratory tests of CS and other D-KEFS measurements

Supplementary Table 2. *CS* tasks - mean normalized results for NTP.

One-Sample Statistics NTP

	N	Mean	Std. Deviation
CS Processing speed	23	104.66	5.09
CS Attention	23	108.93	4.62
CS Learning	23	103.80	9.06
CS Working Memory Speed	23	104.10	6.76
CS Working Memory Accuracy	23	106.01	6.45

Supplementary Table 3. One-sample t-test assessing whether *CS* results of NTP were significantly different from the norm (i.e. 100).

One-Sample Test NTP

	Test Value = 100			
	t	df	Sig.	Mean Difference
CS Processing Speed	4.39	22	0.001	4.66
CS Attention	9.28	22	0.001	8.93
CS Learning	2.01	22	0.057	3.80
CS Working Memory Speed	2.91	22	0.008	4.10
CS Working Memory Accuracy	4.47	22	0.001	6.01

Supplementary Table 4. *CS* tasks- average normalized results for PLP.

One-Sample Statistics PLP

	N	Mean	Std. Deviation
CS Processing Speed	28	107.12	4.96
CS Attention	28	108.71	3.29
CS Learning	28	104.90	8.61
CS Working Memory Speed	28	104.78	6.73
CS Working Memory Accuracy	28	104.37	14.53

Supplementary Table 5. One-sample t-test assessing whether *CS* results of PLP were significantly different from the norm (i.e. 100).

One-Sample Test PLP

	Test Value = 100			
	t	df	Sig.	Mean Difference
CS Processing Speed	7.61	27	0.001	7.12
CS Attention	13.99	27	0.001	8.71
CS Learning	3.01	27	0.006	4.90
CS Working Memory Speed	3.76	27	0.001	4.78
CS Working Memory Accuracy	1.59	27	0.123	4.37

Supplementary Table 6. *D-KEFS* tasks *CWI* and *TMT* - average normalized results for NTP.

One-Sample Statistics National Team Players

	N	Mean	Std. Deviation
CWI 3	23	12.13	2.28
CWI 4	23	12.30	1.66
TMT 2-3	23	13.78	1.68
TMT 4	23	12.17	1.40

Supplementary Table 7. One-sample t-test for assessing whether *CWI* and *TMT* results of NTP were significantly different from the norm (i.e. 100).

One-Sample Test National Team Players

	Test Value = 10			
	t	df	Sig.	Mean Difference
CWI 3	4.48	22	0.001	2.13
CWI 4	6.64	22	0.001	2.30
TMT 2-3	10.81	22	0.001	3.78
TMT 4	7.43	22	0.001	2.17

Supplementary Table 8. *D-KEFS* tasks *CWI* and *TMT* - average normalized results for PLP.

One-Sample Statistics Premier League Players

	N	Mean	Std. Deviation
CWI 3	28	10.89	2.57
CWI 4	28	10.89	2.28
TMT 2-3	28	13.11	1.97
TMT 4	28	11.54	1.62

Supplementary Table 9. One-sample t-test for assessing whether *CWI* and *TMT* results of PLP were significantly different from the norm (i.e. 100).

One-Sample Test Premier League Players

	Test Value = 10			
	t	df	Sig.	Mean Difference
CWI 3	1.84	27	0.077	0.89
CWI 4	2.07	27	0.048	0.89
TMT 2-3	8.35	27	0.001	3.11
TMT 4	5.01	27	0.001	1.54

Supplementary Table 10. Independent sample t-test assessing whether there was a significant difference between *CWI*, *TMT* and CS results of NTP and PLP.

Independent Samples Test

	Levene's Test for Equality of Variances		t-test for Equality of Means			
	F	Sig.	t	df	Sig.	Mean Difference
CWI 3	0.25	0.621	1.80	49	0.078	1.24
CWI 4	**1.55**	**0.219**	**2.47**	**49**	**0.017**	**1.41**
TMT 2-3	0.003	0.958	1.30	49	0.199	0.68
TMT 4	0.98	0.328	1.49	49	0.144	0.64
CS Processing Speed	0.00	0.994	-1.75	49	0.087	-2.47
CS Attention	2.90	0.095	0.20	49	0.844	0.22
CS Learning	0.09	0.765	-0.44	49	0.658	-1.10
CS Working Memory Speed	0.009	0.924	-0.36	49	0.721	-0.68
CS Working Memory Accuracy	1.73	0.195	0.50	49	0.618	1.64

Supplementary Table 11.

Correlation between rated game *intelligence* vs WM (*Working memory / Learning*) and Stroop tasks (*CWI 3* and *4*)

Pearson correlation

	r	Sig.
CS Working Memory Accuracy	0.19	p=0.185
CS Learning	0.12	p=0.382
CWI 3	0.18	p=0.216
CWI 4	0.16	p=0.246
